# GOES-R land surface products at Western Hemisphere eddy covariance tower locations

**DOI:** 10.1038/s41597-024-03071-z

**Published:** 2024-03-07

**Authors:** Danielle Losos, Sophie Hoffman, Paul C. Stoy

**Affiliations:** 1 Department of Biological Systems Engineering, University of Wisconsin – Madison, Madison, WI USA; 2 Department of Atmospheric and Oceanic Sciences, University of Wisconsin – Madison, Madison, WI USA

**Keywords:** Carbon cycle, Ecosystem ecology

## Abstract

The terrestrial carbon cycle varies dynamically on hourly to weekly scales, making it difficult to observe. Geostationary (“weather”) satellites like the Geostationary Environmental Operational Satellite - R Series (GOES-R) deliver near-hemispheric imagery at a ten-minute cadence. The Advanced Baseline Imager (ABI) aboard GOES-R measures visible and near-infrared spectral bands that can be used to estimate land surface properties and carbon dioxide flux. However, GOES-R data are designed for real-time dissemination and are difficult to link with eddy covariance time series of land-atmosphere carbon dioxide exchange. We compiled three-year time series of GOES-R land surface attributes including visible and near-infrared reflectances, land surface temperature (LST), and downwelling shortwave radiation (DSR) at 314 ABI fixed grid pixels containing eddy covariance towers. We demonstrate how to best combine satellite and *in-situ* datasets and show how ABI attributes useful for ecosystem monitoring vary across space and time. By connecting observation networks that infer rapid changes to the carbon cycle, we can gain a richer understanding of the processes that control it.

## Background & Summary

The terrestrial carbon cycle responds to environmental variability, ecological processes like succession, and anthropogenic management at time scales from millennia (or longer^1^) to minutes (or shorter^2^). Extreme events^3,4^, phenological shifts^5^, and land management can impact ecosystem carbon cycling on sub-daily time scales, as can variability in solar radiation which largely determines daily carbon dioxide flux if other environmental factors are not limiting^6,7^. These limitations can emerge dynamically as the day progresses if, for example, high temperatures^8^, vapor pressure deficit^9^, or plant hydrologic limitations^10^ induce stomatal closure or compromise the photosynthetic machinery. Observing the carbon cycle and the variables that affect it over the time scales at which they covary is critical for understanding the dynamics of the earth system.

Polar-orbiting satellites have return intervals on time scales of days or longer to create data products with time steps of days to years^11,12^, limiting our ability to observe the land surface on sub-daily intervals^13^. Geostationary satellites provide real-time observations on time scales of minutes (or less^14^), and many now host radiometers that measure at visible and infrared wavelengths^15,16^ which are key for understanding carbon cycle processes^13,17^. These shortwave reflective bands allow us to track key variables including the normalized difference vegetation index (NDVI) commonly used to estimate the leaf area index^17–19^, and the near infrared reflectance vegetation^20^ (NIRv; NDVI multiplied by infrared reflectance) which is strongly linked to ecosystem carbon uptake via gross primary productivity (GPP, refs. ^21–23^), especially when multiplied by incident radiation to derive the NIRvP (ref. ^21^). Geostationary satellite data have also long been used to estimate terrestrial evapotranspiration^24^ to which the carbon cycle is coupled^25^, and to create products for key variables that drive carbon cycle processes including downwelling shortwave radiation (DSR) (refs. ^26–28^) and land surface temperature (LST) (refs. ^29,30^).

Information from the Geostationary Environmental Operational Satellite - R Series (GOES-R) satellites is created and distributed by the U.S. National Oceanic and Atmospheric Administration (NOAA) in near-real time for weather forecasting and public awareness of critical meteorological events. The ecosystem-atmosphere exchange of carbon dioxide is commonly measured using the eddy covariance technique to create time series of variables like GPP that can extend months, years, or decades into the past. Most eddy covariance towers are managed by individual laboratories as opposed to a coordinated entity, and therefore contain measurements that are often processed and published according to the personal schedule of each tower manager. This makes it difficult for eddy covariance towers to deliver real-time information. In other words, there is a temporal mismatch between eddy covariance and geostationary satellite data dissemination that needs to be addressed in order to link these rich time series. In addition, the file structures of these two data records are not inherently compatible. While the flux tower observations are time series by nature, GOES-R data are cataloged as individual raster image files, with hundreds of new files produced every day. To convert a stack of images into a single-pixel time series, every file at each respective timestamp must be read to extract the observation at that one pixel.

The purpose of the present analysis is to bridge this gap by creating time series from GOES-R data products at 314 eddy covariance tower locations from the AmeriFlux and NEON tower networks^31,32^. By providing geostationary satellite data in the same format, file type, and time step as eddy covariance data, we hope that the flux community finds benefit from satellite data and the geostationary satellite community finds new ways to create products of interest to land surface science. We first describe the Advanced Baseline Imager (ABI) and key land surface products generated by NOAA from the imagery, then explain the AmeriFlux and NEON networks and their data structure. The ABI land surface products that we organize include Cloud and Moisture Imagery (CMI), Bidirectional Reflectance Factors (BRF), Land Surface Albedo (LSA), Downward Shortwave Radiation (DSR), Land Surface Temperature (LST), Clear Sky Mask, Aerosol Detection, and Aerosol Optical Depth (AOD) (Table 2). We then provide examples of how to best connect “hypertemporal” observations of land surface attributes from satellites with time series of micrometeorological observations including eddy covariance measurements of carbon dioxide flux between ecosystems and the atmosphere.

## Methods

### The GOES-R series advanced baseline imager (ABI)

The ABI is the primary Earth-observing sensor aboard GOES-R^15,16^. The four satellite GOES-R Series began in November 2016 with the launch of GOES-16. GOES-16 has remained in the GOES-East position ever since. GOES-17 served as GOES-West starting in 2018; however, a cooling issue on its loop heat pipe caused partial loss of imagery, and it was replaced by GOES-18 in 2022 (ref. ^33^). The final satellite in the GOES-R Series is scheduled to launch in 2024 after which point a new series of satellites will be launched by the GeoXO mission^34^. GOES-East and GOES-West orbit at approximately 36,000 kilometers above the equator at 75.2 and 137.2 degrees West. Together they view most of the Western Hemisphere, from eastern Africa to Australia and from Alaska to Chile^35^.

The ABI is a passive radiometer that scans the atmosphere, oceans, and Earth surface at sixteen discrete wavelengths ranging from visible to thermal infrared. In its current operational mode (Mode 6), the ABI produces a full disk hemispherical image every ten minutes, a CONUS (Continental United States) or PACUS (Pacific U.S.) image every five minutes, and two mesoscale images per minute. Mesoscale regions are small movable domains that can provide detailed temporal coverage of regions with heightened meteorological interest^16^. Twelve of the sixteen ABI bands have 2 kilometer (km) spatial resolution at the sub-satellite point (nadir). The shortwave bands 1, 3 and 5 have 1 km resolution, while band 2 has 0.5 km resolution (see Table S2)^35^.

### ABI Fixed Grid

**Fig. 1:**
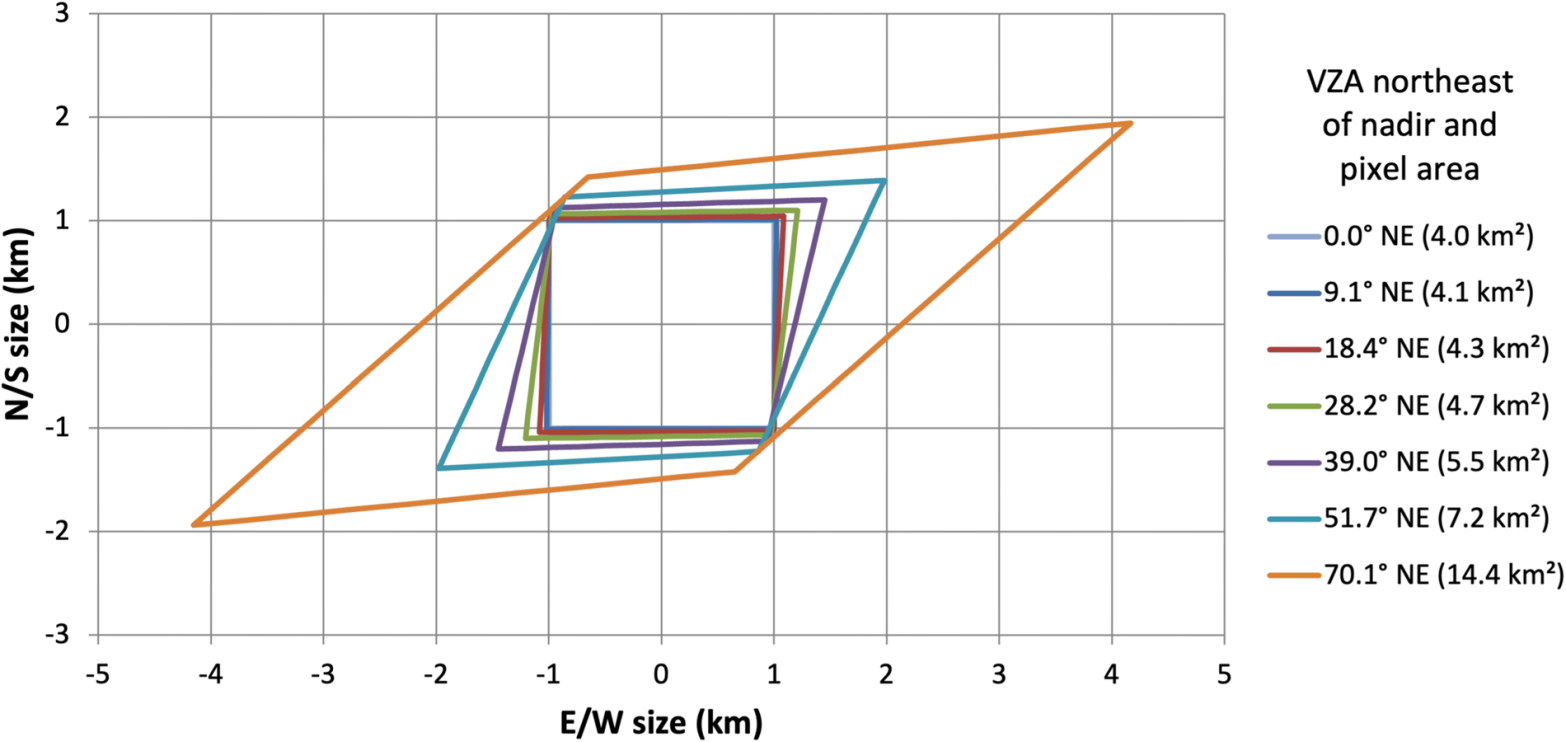
GOES-R ABI pixel outlines and areas (in km/km^2^) at increasing view zenith angle (VZA) northeast of nadir (in degrees). The pixel size and VZA relationship and this figure were developed by Randall Race.

Due to the geostationary orbit of GOES satellites, their position and viewing geometry relative to the Earth’s surface is, ideally, unchanging. The ABI fixed grid represents each spatial domain (full disk, CONUS/PACUS, and mesoscale) as a grid of ABI scan angles which describe the North/South and East/West orientation of the ABI scan mirrors for every pixel. For each spatial resolution, any two adjacent pixels have equal angular separation. In other words, scan angles remain constant across the fixed grid^16^. However, pixel surface area increases moving away from nadir because a constant scan angle corresponds with greater distance as the earth curves away from the sub-satellite point. While a 2-km GOES-East ABI pixel is 4 km^2^ at nadir, the pixel area stretches to 7.2 km^2^ near Madison, Wisconsin and 14.3 km^2^ near Seattle, Washington (near the furthest extent of the L2 BRF product for GOES-16) as demonstrated in Fig. 1.

To accurately map eddy covariance tower locations onto the ABI fixed grid and obtain ABI observations, we needed to align the ABI and tower location information. For GOES-R Level 1b and most Level 2 products, geographic information for each data file is stored as horizontal (x) and vertical (y) scan angles. Converting tower geodetic latitude and longitude coordinates to ABI scan angle coordinates is necessary, as described in the equations in the Supplementary Information (S1–S7). The following Earth model constants are defined by the Geodetic Reference System 1980 (GRS 80) ellipsoid: the Earth’s semi-major axis (r_eq_), semi-minor axis (r_pol_) and eccentricity (e, ref. ^36^). The satellite’s longitude (λ_0_) is constant, while targets on the Earth’s surface are described by their longitude (λ), latitude (φ), and elevation (z) (ref. ^36^). For many earth science applications, the opposite conversion–scan angles to geodetic coordinates–is necessary to geolocate pixels on the Earth surface (Supplementary Information S8–S15).

**Fig. 2:**
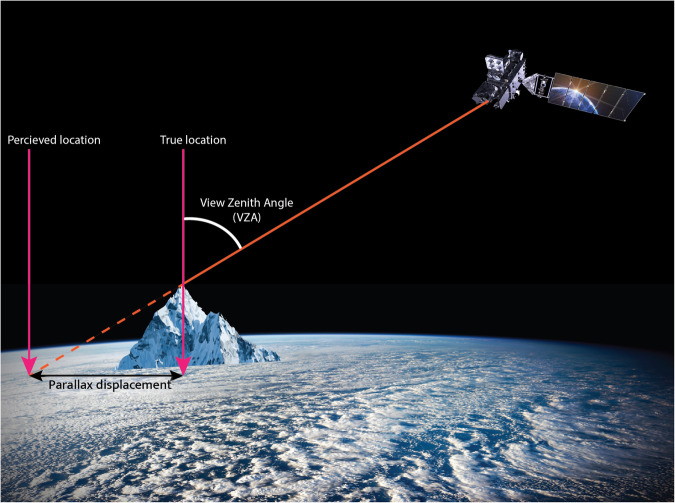
A schematic showing how off-nadir view angles can impact the projected locations of elevated surface targets^49^.

ABI fixed grid products are not terrain-corrected: there is no adjustment for the off-nadir view angle of the satellite relative to surface targets. The “parallax effect” causes the satellite to perceive high-elevation targets to be displaced from their true location^37^ by a distance that increases with target’s elevation and satellite view zenith angle (VZA) as described in Fig. 2. GOES satellites only have a nadir view of equatorial surface targets at the sub-satellite points (75.2 °W and 137.2 °W); all other regions require terrain-correction for proper geolocation of elevated targets. Since the present research is concerned with the eddy covariance towers at point locations, it is only necessary that the correct ABI pixel is matched with the targeted tower. The true tower location is shifted by the magnitude and direction of the parallax displacement to the location where it is perceived to be by the ABI fixed grid (see “Corrected Lat/Lon” in Table 1), before the tower is matched with an ABI pixel.

**Table 1:** For each eddy covariance tower location the following constant variables are recorded, categorized as Ameriflux site characteristics, viewing geometry and measurements from GOES-R, and ecological information.

Constant Variables	Variable Name	Description	Units
**Site Id**	SITE_ID	Name identification of AmeriFlux site.	N/A
**Time zone**	TIMEZONE	Time zone abbreviation and UTC offset from local time.	TTT + 0
**Geodetic coordinates**	SITE_LAT, SITE_LON	AmeriFlux-provided latitude and longitude of site.	Degrees
**Elevation**	ELEVATION	AmeriFlux-provided elevation of the site.	Meters
**ABI pixel area**	PIXEL_AREA	Surface area of ABI pixel where the AmeriFlux tower resides.	Square kilometers
**Terrain-corrected geodetic coordinates**	CORRECTED_LAT, CORRECTED_LON	False latitude and longitude adjusted to account for parallax displacement.	Degrees
**View Azimuth Angle**	VAA	Horizontal angle between the line connecting the satellite to the surface and a ray from the site to polar north.	Degrees
**View Zenith Angle**	VZA	Angle between the line connecting the satellite to the surface, and the tangent normal to the surface.	Degrees
**Parallax displacement**	PARALLAX	Displacement of the target location as perceived by the satellite due to off-nadir VZA.	Meters
**Vegetation type (IGBP)**	VEGETATION_IGBP	International Geosphere Biosphere Programme (IGBP) Type 1 land cover scheme identifies 17 land cover classes (0 – 16) which includes 11 natural vegetation classes, 3 developed and mosaicked land classes, and three non-vegetated land classes^74^.	N/A
**Climate class (Köppen)**	CLIMATE_KOEPPEN	Classification that divides terrestrial climates into five major types based on seasonal precipitation and temperature patterns. Represented by the letters A, B, C, D, and E.	N/A

Calculating the perceived tower location takes advantage of the aforementioned conversion from tower geodetic coordinates to ABI scan angles. Typically, this conversion assumes that the geocentric distance (r_c_) between the center and the surface of the Earth is equal to the modeled earth radius in GRS 80 (ref. ^38^). In addition, our calculation accounts for increased geocentric distance to a target above sea-level. Therefore, the eddy covariance tower site’s elevation (z, see ref. ^39^) is added to r_c_.

### ABI Level 2 (L2) products

The ABI scans the full disk in under ten minutes, data are processed, and individual netCDF (.nc) files for each data product are made available in near-real time. Most ABI products are created every time a full-disk and CONUS scan is completed, but others currently have less frequent refresh rates, such as once per hour^16^. Here we describe the ABI products that we have compiled for flux tower locations.

### L2 Cloud and moisture imagery (CMI)

The CMI product provides reflectance values or brightness temperatures at sixteen ABI channels. The primary data source for this product is the Level 1b (L1b) Radiance product, measuring solar radiation (in W m^−2^ sr^−1^) at all sixteen ABI bands^35^. We used the Multiband CMI product which resamples all 16 bands to a uniform 2 km grid, despite the higher native spatial resolutions of select bands (Table S1). For the six reflective bands (bands 1– 6), radiance values are converted to a dimensionless reflectance factor ranging from 0 to 1.3 by multiplying by the incident Lambertian equivalent radiance (κ)

1 $${\rm{\kappa }}=\frac{{{\rm{\pi d}}}^{2}}{{{\rm{E}}}_{{\rm{sun}}}}$$

where *d* is the instantaneous Earth-Sun distance in Astronomical Units and *E*_*sun*_ is the solar irradiance in the respective bandpass (W m^−2^ *μ*m^−1^) as described in the GOES-R Product User Guide (PUG) Volume 5 (ref. ^36^).

CMI reflectances are considered top-of-atmosphere (TOA) rather than surface reflectances because they measure the total reflectance received by the satellite at the top of the atmosphere, without accounting for atmospheric scattering. For the ten emissive bands (7–16), L1b radiances are converted to brightness temperature (K) using Planck’s function^36^. These longer wavelength measurements provide critical atmospheric and environmental context such as characterizing clouds, aerosols, fire, and snow that are of importance for terrestrial carbon cycle science^37^.

### Bidirectional reflectance factors (BRF)

The BRF product has been an operational ABI product since August 18, 2021, and provides surface reflectances for ABI bands 1, 2, 3, 5, and 6 as a byproduct of the LSA product^38^. The BRF product does not include ABI band 4 (the “cirrus” band) because this channel at 1.37 μm absorbs water vapor, thus it is not an atmospheric window and does not convey surface information^37^. The descriptor *bidirectional* aptly explains that these reflectance factors are affected by both the solar incidence angle on the land surface as well as the viewing angle of the ABI sensor. Due to the unchanging position of the geostationary satellite, BRF values at a site vary throughout the day as a function of the changing solar position in relation to the constant sensor viewing angle. The LSA algorithm derives Bidirectional Reflectance Distribution Function (BRDF) parameters, which are used to both estimate broadband albedo and to simulate surface reflectance on cloudy days when it cannot be measured directly. Solving for BRDF parameters is accomplished by minimizing a cost function which relates TOA reflectances and AOD (ref. ^38^), both of which can be computed from ABI measurements over the course of the day as the solar zenith angle changes.

The BRF algorithm has two paths available for deriving surface reflectances, depending on whether clear-sky observations are available. The default and more accurate method, the R3 algorithm, assumes the surface is Lambertian and directly calculates surface reflectance (*r*_*s*_) from TOA reflectances (*r*) and atmospheric parameters^39^. Transmittance (γ), path reflectance (*r*_*0*_), and spherical albedo (*ρ*) are retrieved from a look-up table which pre-calculates these parameters given viewing geometry and AOD using the radiative transfer model MODTRAN^40^ (Eq. 2).

2 $${{\rm{r}}}_{{\rm{s}}}=\frac{{\rm{r}}-{{\rm{r}}}_{0}}{{\rm{\gamma }}+\left({\rm{r}}-{{\rm{r}}}_{0}\right){\rm{\rho }}}$$

3 $${\rm{BRF}}={\rm{\pi BRDF}}$$

A back-up method is necessary for cloudy conditions where the atmospheric parameters are not available. The R2 algorithm is used to calculate surface BRF from the BRDF parameters retrieved from the prior day’s TOA reflectance measurements (Eq. 3) to model BRF throughout the day given satellite and solar viewing geometries. Suggested dataset usage for the BRF product is provided in *Usage Notes*.

### Land surface albedo (LSA)

The Land Surface Albedo (LSA) product is produced in harmony with the BRF land surface reflectance product. Instantaneous broadband albedo is ideally derived from the clear-sky TOA reflectances and the prior day’s BRDF parameters, which in turn are estimated from aerosol optical depth, a daily stack of shortwave reflectances, and albedo climatology^38^.

### Downward shortwave radiation (DSR)

The DSR product measures the total instantaneous shortwave irradiance incident at the Earth’s surface integrated over visible and infrared wavelengths (0.2 to 4.0 μm, ref. ^28^) at the time of measurement (the top of the hour). DSR consists of both direct and diffuse solar radiation, attenuated and scattered by the atmosphere, in W m^−2^. The DSR product is currently produced just once per hour at full disk and CONUS domains. DSR is calculated from surface albedo and atmospheric conditions using two different modes of retrieval. The “direct” path determines atmospheric conditions from lookup tables using inputs of ABI L2 surface and atmospheric products. When these products are unavailable, the “indirect path” uses a 28-day aerosol climatology and lookup tables to estimate instantaneous surface albedo and atmospheric conditions^36^. A unique aspect of this L2 product is that DSR data is projected onto a Global Latitude and Longitude Grid, rather than the ABI Fixed Grid used for all other products discussed here (see *Converting between projections* in the Supplementary Information).

### Land surface temperature (LST)

The Land Surface (Skin) Temperature (LST) product records the instantaneous temperature of the Earth’s surface in Kelvin^41,42^. The LST product can only be produced under clear-sky conditions, hence cloud-obstructed observations are masked out. The equation for calculating LST depends on the emissivities and brightness temperatures at the ABI thermal bands at 11.19 µm, 12.27 µm, and the “split-window” difference between these brightness temperatures and emissivities, as well as coefficients that depend on day/night and atmospheric conditions^41^. Like DSR, LST is also produced just once per hour. For this reason, LST and DSR were upsampled to match the half-hourly cadence of most AmeriFlux time series and noted in the data files. Half-hourly LST and DSR values were estimated using cubic interpolation between consecutive existing LST and DSR observations. However, this interpolation is limited to instances where both observations on the hour are known; gaps where an hour or more is missing are not filled.

### Clear sky mask

The Clear Sky Mask, also called the Cloud Mask, provides a binary image with each pixel classified as either “clear” or “cloudy”^43^. First, the algorithm employs spectral, spatial and temporal tests on each pixel to categorize the pixel as “clear”, “probably clear”, “probably cloudy” and “cloudy.” Classifications are compared to the model outputs from the Community Radiative Transfer Model (CRTM, ref. ^44^). The four-class Cloud Mask intermediate product is a critical input to many other ABI L2 product algorithms; however, the four classes are condensed into a binary mask before the final product is distributed to users.

### Aerosol detection

The Aerosol Detection product consists of three separate variable layers, each of which is a binary mask representing ‘yes detection’ or ‘no detection’^45^. The three types of aerosol detections are dust, smoke, and aerosols generally (when either dust or smoke has been detected). There are two distinct algorithm pathways for observations over land and ocean, but both begin by masking out high and optically thick clouds, and proceed with a series of threshold tests on the reflective and thermal ABI bands that detect and characterize aerosols. Notably, an Aerosol Detection product data quality flag denotes “invalid detection due to snow_ice_clouds”, information retrieved from the GOES L2 Snow/Ice product, which can be used as a proxy for masking out snow surface cover in other products.

### Aerosol optical depth (AOD)

The AOD product retrieves aerosol optical thickness over both land and ocean, using different retrieval pathways^46,47^. Specifically, AOD measures the extinction of solar radiation due to atmospheric aerosols at a wavelength of 550 nm. In addition, the product provides the aerosol particle size, as represented by two Ångström exponents. The algorithm determines AOD by comparing instantaneous TOA reflectance measurements to modeled reflectances under a range of aerosol values and atmospheric conditions, precalculated using a radiative transfer model and stored in a look-up table^47^. Different ABI reflectance channels are used for the land and the ocean AOD retrievals. The AOD algorithm relies on the aerosol type characterization generated by the Aerosol Detection product.

### ABI fixed grid

Due to the geostationary orbit of GOES satellites, their position and viewing geometry relative to the Earth’s surface is, ideally, unchanging. The ABI fixed grid represents each spatial domain (full disk, CONUS/PACUS, and mesoscale) as a grid of ABI scan angles which describe the North/South and East/West orientation of the ABI scan mirrors for every pixel. For each spatial resolution, any two adjacent pixels have equal angular separation. In other words, scan angles remain constant across the fixed grid^16^. However, pixel surface area increases moving away from nadir because a constant scan angle corresponds with greater distance as the earth curves away from the sub-satellite point. While a 2-km GOES-East ABI pixel is 4 km^2^ at nadir, the pixel area stretches to 7.2 km^2^ near Madison, Wisconsin and 14.3 km^2^ near Seattle, Washington (near the furthest extent of the L2 BRF product for GOES-16) as demonstrated in Fig. 1.

To accurately map eddy covariance tower locations onto the ABI fixed grid and obtain ABI observations, we needed to align the ABI and tower location information. For GOES-R Level 1b and most Level 2 products, geographic information for each data file is stored as horizontal (x) and vertical (y) scan angles. Converting tower geodetic latitude and longitude coordinates to ABI scan angle coordinates is necessary, according to the equations in *Converting Projections* (Equations A1 - A7). The following Earth model constants are defined by the Geodetic Reference System 1980 (GRS 80) ellipsoid: the Earth’s semi-major axis (r_eq_), semi-minor axis (r_pol_) and eccentricity (e, ref. ^48^). The satellite’s longitude (λ_0_) is constant, while targets on the Earth’s surface are described by their longitude (λ), latitude (φ), and elevation (z) (ref. ^48^). For many earth science applications, the opposite conversion – scan angles to geodetic coordinates – is necessary to geolocate pixels on the Earth surface (Equations A8 - A15).

ABI fixed grid products are not terrain-corrected: there is no adjustment for the off-nadir view angle of the satellite relative to surface targets. The “parallax effect” causes the satellite to perceive high-elevation targets to be displaced from their true location^49^ by a distance that increases with target’s elevation and satellite view zenith angle (VZA) as described in Fig. 2. GOES satellites only have a nadir view of equatorial surface targets at the sub-satellite points (75.2 °W and 137.2 °W); all other regions require terrain-correction for proper geolocation of elevated targets. Since the present research is concerned with the eddy covariance towers at point locations, it is only necessary that the correct ABI pixel is matched with the targeted tower. The true tower location is shifted by the magnitude and direction of the parallax displacement to the location where it is perceived to be by the ABI fixed grid (see “Terrain-corrected geodetic coordinates” in Table 1) before the tower is matched with an ABI pixel.

Calculating the perceived tower location takes advantage of the aforementioned conversion from tower geodetic coordinates to ABI scan angles. Typically, this conversion assumes that the geocentric distance (r_c_) between the center and the surface of the Earth is equal to the modeled earth radius in GRS 80 (ref. ^36^). In addition, our calculation accounts for increased geocentric distance to a target above sea-level. Therefore, the eddy covariance tower site’s elevation (z, see ref. ^50^) is added to r_c_.

### Calculating NIRvP using GOES-R

To calculate NDVI, NIRv and NIRvP on a per-pixel basis, the three inputs required are ABI band 2 (red) surface reflectance, ABI band 3 (NIR) surface reflectance, and DSR, all resampled to 2-km spatial resolution. These values are retrieved from the L2 BRF and DSR products, respectively, and observations are filtered to remove poor quality observations using the corresponding data quality flags. The NDVI is the normalized difference between the red and NIR (Eq. 4), which is multiplied by NIR to derive NIRv (Eq. 5) and is then multiplied by photosynthetically active radiation (PAR) to derive NIRvP (Eq. 6); both NIRv and NIRvP are strongly related to GPP (refs. ^20,51^). Forthcoming NOAA GOES-R product releases include a photosynthetically active radiation (PAR) product and additionally a collaborative effort ‘GeoNEX’ is working to provide global gridded PAR and DSR from multiple geostationary satellites^52^. In the interim, we estimated PAR (in W m^−2^) as 0.45 times DSR (refs. ^53,54^); we note that this will induce a small amount of uncertainty into the final NIRvP estimate as this conversion factor varies depending on atmospheric composition and solar position^53,55,56^.

4 $${\rm{NDVI}}=\frac{{\rm{NIR}}-{\rm{Red}}}{{\rm{NIR}}+{\rm{Red}}}$$

5 $${\rm{NIRv}}={\rm{NDVI}}\times {\rm{NIR}}$$

6 $${\rm{NIRvP}}={\rm{NIRv}}\times {\rm{PAR}}$$

We note that some implementations of NIRv subtract a factor, often 0.08, to account for soil reflectance^52^. Adjustment factors can be added to the calculation of (Eq. 5) based on soil characteristics of a given study region with the data provided. The flux community often uses photosynthetically active photon flux density with typical units of μmol m^−2^ s^−1^. PAR can be converted to photosynthetically active photon flux density PPFD by using a conversion factor of approximately 4.56 μmol J^−1^ (ref. ^57^).

### Eddy covariance

**Fig. 3:**
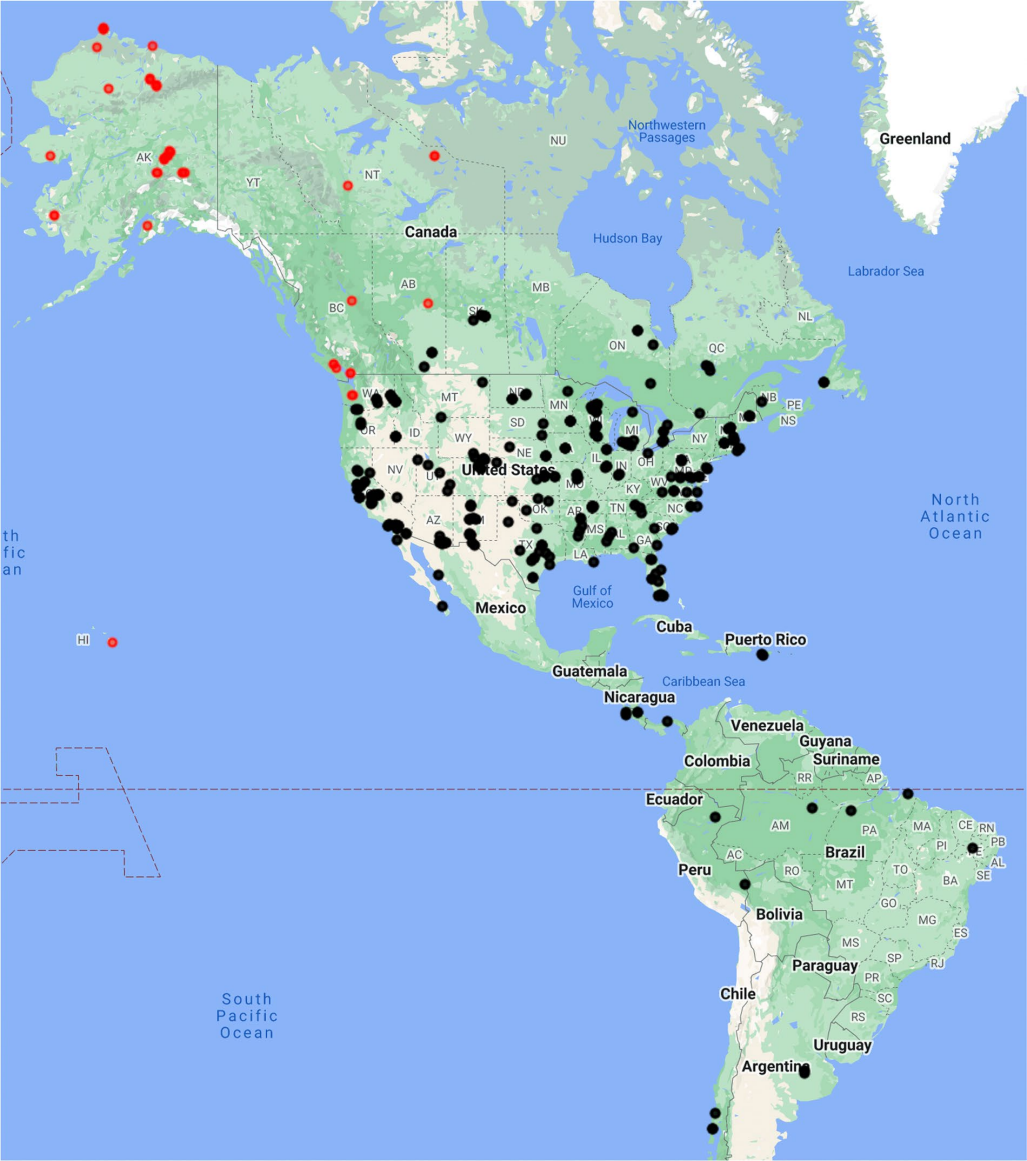
AmeriFlux and NEON, Inc. eddy covariance sites where GOES-16 L2 Bidirectional Reflectance Factor (BRF) products are available as black dots and those sites with view zenith angles (VZA) > 70° (and therefore without available data) as red dots, produced in Google Earth Engine^75^.

The AmeriFlux network relies on the efforts of individual tower-operating teams across the Western Hemisphere^31^ which, coupled with NEON, Inc. eddy covariance towers, resulted in 314 eddy covariance towers at VZA under 70° with publicly-available data at time of writing^50^ (Fig. 3) These data are collected by the tower-operating teams or NEON, Inc.^32,58^ and provide half-hourly (or in rare instances hourly) sums of carbon dioxide, water, sensible heat, and/or other trace gas fluxes and half-hourly (or hourly) averages or sums of micrometeorological variables, all quality control-checked by common algorithms^59,60^ and organized as .csv files. These files are updated shortly after new data are uploaded to AmeriFlux or NEON, which in practice may result in delays that can extend from months to years from the time at which data were collected.

## Data Records

We created 314 .csv files of GOES-R time series at eddy covariance tower locations (Fig. 3, Table S2) on the same half-hourly interval as most eddy covariance observations. Each of these .csv files as well as a table containing site information for all 314 AmeriFlux sites are available at the Environmental Data Initiative (EDI) Data Portal: 10.6073/pasta/c3bb20a62edbf8548cbb30e79a689a5b^61^. To do so, we extracted surface reflectances, cloud and aerosol products, LST and DSR – and associated data quality control flags – from GOES-R files at the 314 eddy covariance tower locations as described in Tables 1 and 2 for the January 2020 - December 2022 period for which the GOES-R L2 products are available. The CMI product is available dating back to 2017, and these longer time series are available upon request. Files also include satellite-earth geographic information including view and solar zenith angles and parallax-adjusted geographic coordinates as well as time information in standard UTC units and local standard time, the latter of which is the convention for the eddy covariance data files. See *EDI data package summary* in the Supplementary Information for more detail.

**Table 2:** The names, descriptions, and units for the variables in the primary data record that change dynamically at each timestep.

Dynamic variables	Variable Name	Data Quality Flag	Description	Units
**Aerosol Detection**	ADP_aero	ADP_DQF	Binary mask that signals the presence of any aerosols in the pixel.	Unitless factor, 0 to 1
	ADP_smk		Binary mask that signals the presence of smoke aerosols in the pixel.	
	ADP_dust		Binary mask that signals the presence of dust aerosols in the pixel.	
**Aerosol Optical Depth**	AOD	AOD_DQF	The extinction of solar radiation due to atmospheric aerosols at a wavelength of 550 nm.	Dimensionless quantity
**Bidirectional Reflectance Factor**	BRF1, BRF2, BRF3, BRF5, BRF6	BRF_DQF	Surface reflectance, the ratio between outgoing radiance at one given direction and incoming radiance at another given direction, at the Earth’s surface for five of the six ABI reflective bands (1–3, 5, 6).	Unitless factor from 0 to 1
**Clear Sky Mask**	ACM	ACM_DQF	Binary mask indicating a medium or high probability of cloud in the pixel.	Unitless factor, 0 to 1
**Cloud and Moisture Imagery**	CMI_C01, CMI_C02, CMI_C03, CMI_C04, CMI_C05, CMI_C06	DQF_C01, DQF_C02, DQF_C03, DQF_C04, DQF_C05, DQF_C06	Top-of-atmosphere (TOA) reflectances at the six ABI reflective bands (1 - 6). Reflectance is the ratio between outgoing radiance at one given direction and incoming radiance at another given direction.	Unitless factor from 0 to 1
	CMI_C07, CMI_C08, CMI_C09, CMI_C10, CMI_C11, CMI_C12, CMI_C13, CMI_C14, CMI_C15, CMI_C16	DQF_C07, DQF_C08, DQF_C09, DQF_C10, DQF_C11, DQF_C12, DQF_C13, DQF_C14, DQF_C15, DQF_C16	TOA brightness temperatures for the 10 ABI emissive bands (7–16). Brightness temperature is the temperature of a theoretical blackbody emitting the equivalent intensity of radiation at the specified wavelength.	Kelvin
**Downward Shortwave Radiation**	DSR	DSR_DQF	Instantaneous total shortwave irradiance (flux) received at the Earth’s surface integrated over the 0.2 to 4.0 m wavelength interval.	W/m^2^
**Land Surface Albedo**	LSA	LSA_DQF	Ratio between outgoing and incoming shortwave irradiance from 0.4 to 3.0 m (broadband) at the Earth’s surface.	Unitless factor, 0 to 1
**Land Surface Temperature**	LST	LST_DQF	Instantaneous land surface skin temperature.	Kelvin
**Normalized Difference Vegetation Index**	NDVI	N/A	Normalized difference between red and near-infrared reflectance (ABI bands 2 and 3).	Unitless factor from −1 to 1
**Near Infrared Reflectance of Vegetation**	NIRv	N/A	NDVI multiplied by near-infrared reflectance (ABI band 3).	Unitless factor from −1 to 1
**Photosynthetically Active Radiation**	PAR	N/A	Approximated by multiplying DSR by 0.45.	W/m^2^
**NIRv multiplied by PAR**	NIRvP	N/A	NIRv multiplied by incoming sunlight (PAR).	W/m^2^
**Solar Azimuth Angle**	SAA	N/A	Horizontal angle between a ray from the site to polar north, and the solar ray.	Degrees
**Solar Zenith Angle**	SZA	N/A	Vertical angle between a tangent normal to the site surface, and the solar ray.	Degrees
**Solar Position**	SOLAR_POS	N/A	Unique solar position defined as the SZA and SAA sum.	Degrees
**UTC timestamp**	UTC_TIME	N/A	The observation time in Coordinated Universal Time (UTC).	yyyy-mm-dd hh:mm:ss.ms
**Local timestamp**	LOCAL_TIME	N/A	The observation time in local time relative to where the AmeriFlux tower site is located.	yyyy-mm-dd hh:mm:ss.ms
**Day of year**	DOY	N/A	Julian day from 0 to 365 (or 366 during Leap Years)	Unitless
**Hour**	HOUR	N/A	Hour of day (0 to 23)	Unitless

The variables are grouped as follows in the table: GOES-R ABI products with associated data quality flags, derived indices from the ABI products, solar geometry, and time information.

**Fig. 4:**
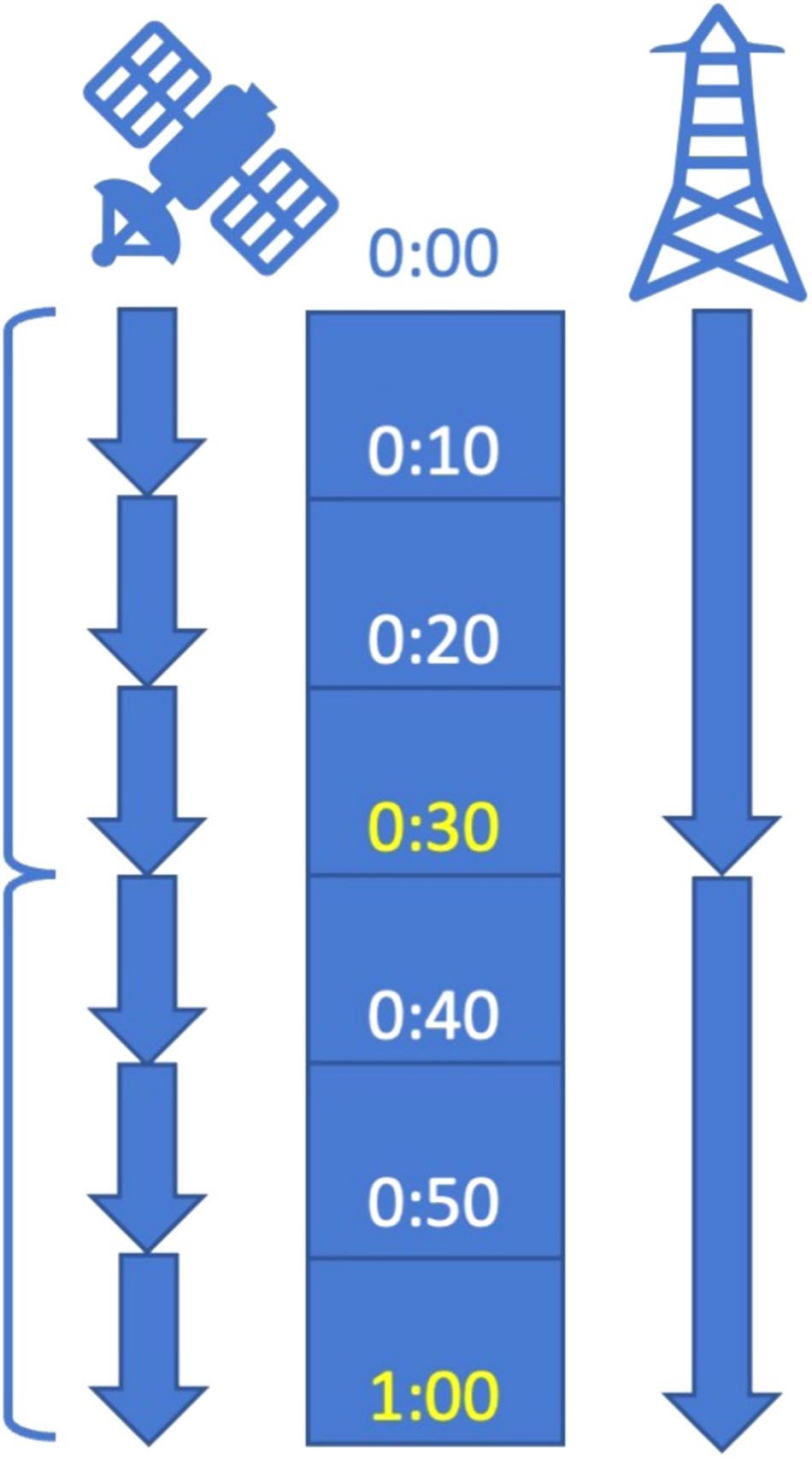
A description of GOES-R Mode 6 full disk timing from the top of the hour (left) that makes a discrete measurement for each pixel scanning from north to south, versus half-hourly eddy covariance data (right) that represent the average or sum of variables measured between a start and end point at the top of the hour and each half hour (or in rare cases hour). The timestamp label for the half-hourly data records is highlighted in yellow.

Linking half-hourly averages of micrometeorological variables and surface-atmosphere fluxes with GOES-R full disk scans presents a challenge. Half-hourly eddy covariance data files utilize the entire thirty minutes of tower measurements, starting at the top of the hour, such that observation midpoints are at 15 and 45 minutes. GOES-R scans begin near the top of the hour, then ten, twenty, thirty, forty and fifty minutes afterward for observations that take approximately ten minutes to complete from north to south (Fig. 4). In other words, there is not a GOES-R scan that aligns cleanly with the midpoint of the eddy covariance observations. For this reason, we collected all three GOES-R scans for each half-hour and calculated average values across the half-hour period. This time alignment effectively makes each tower and GOES-R data record representative of average conditions over the half-hour, matched to the timestamp at the end of the half-hour period. Furthermore, not all ABI products are produced as frequently as the scan cadence: DSR and LST are produced just once per hour when ABI scans at the top of the hour. This observation represents the instantaneous condition, or snapshot, at the scan time. We upsampled DSR and LST observations to match the half-hour eddy covariance interval by performing cubic interpolation between the hourly data points as noted in *Methods*. Users should be aware that values for DSR and LST at the half-hour, or thirty-minute mark, have been estimated through interpolation and are not direct measurements.

## Technical Validation

**Fig. 5:**
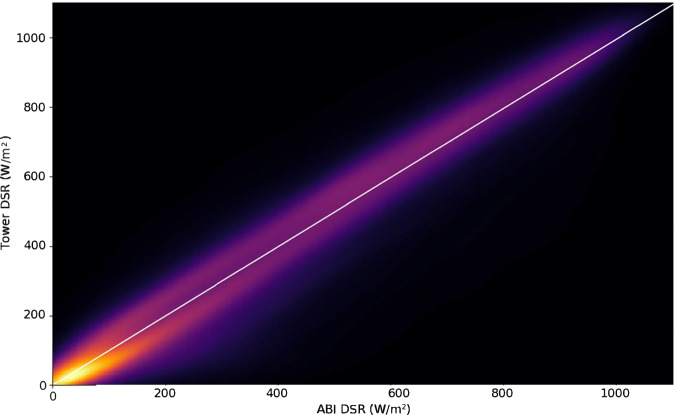
A two-dimensional kernel density (“heat map”) representation of downwelling shortwave radiation (DSR) from the Advanced Baseline Imager (ABI) onboard GOES-R versus eddy covariance tower incident shortwave radiation observations (‘Tower DSR’) for 314 eddy covariance tower sites.

**Fig. 6:**
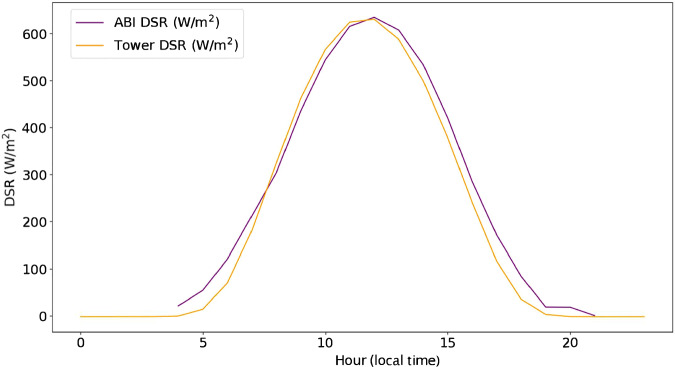
The diurnal course of downwelling shortwave radiation (DSR) from the Advanced Baseline Imager (ABI) onboard GOES-R versus eddy covariance tower incident shortwave radiation observations (‘Tower DSR’) for 314 eddy covariance towers.

We first demonstrate the relationship between tower-measured and GOES-estimated DSR. The ABI DSR product and tower DSR have a strong positive correlation with r^2^ = 0.845. When ABI and tower-measured DSR are plotted against each other (Fig. 5), a faint hysteresis (hole-like feature in the scatterplot) appears at low to middle DSR values. A slight time lag appears to be the cause, resulting in the tower DSR declining before the ABI DSR in the afternoon (Fig. 6). A reason for this discrepancy may be the observation cadence– the ABI sensor measures instantaneous DSR while the eddy covariance towers average the radiation conditions over half-hourly measurement windows.

We further validate the accuracy of GOES-R data by comparing LST estimates between the ABI product and tower measurements. LST can be estimated from tower-measured longwave incoming radiation (LW_IN_) and outgoing radiation (LW_OUT_) according to Eq. 7, proposed by Thakur *et al*., 2022 (ref. ^62^), assuming the emissivity (*ε*) of vegetation is 0.98 (ref. ^62^), and with the Stefan- Boltzmann constant (*σ*) of 5.67 × 10^−8 ^W m^−2^ K^−4^.

7 $${\rm{LST}}=\sqrt[4]{\frac{L{W}_{IN}}{\sigma }-\frac{L{W}_{IN}}{\varepsilon \sigma }+\frac{L{W}_{OUT}}{\varepsilon \sigma }}$$

**Fig. 7:**
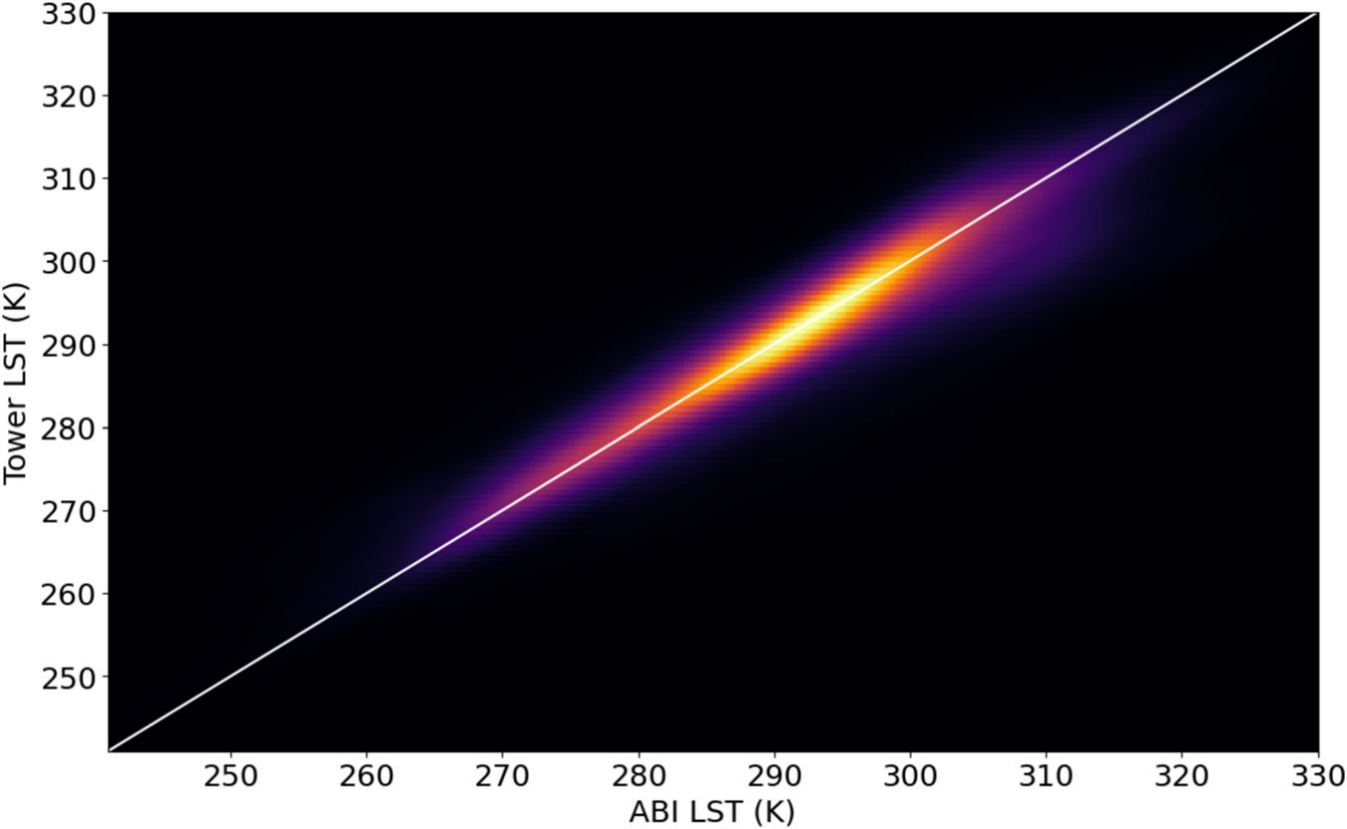
A heatmap depiction of ABI Land Surface Temperature (“ABI LST”) measured in Kelvin (K) plotted against LST derived from eddy covariance tower measurements (“Tower LST”) for 314 eddy covariance tower sites.

Similar to DSR, tower-derived LST and the ABI LST product correlate strongly with r^2^ = 0.844 (Fig. 7) that was similar to when we used a revised approach for calculating surface temperature that incorporates reflected longwave radiation^63^. From this analysis, the offset of GOES and tower-measured LST is likely due in part to ongoing challenges in estimating ecosystem emissivity.

While DSR estimates are expected to be similar between an eddy covariance tower and a 2-km ABI pixel (and to a lesser degree LST), the same cannot be said for GPP. The turbulent flux footprint of an eddy covariance tower is variable and unlikely to align exactly with a 2-km ABI pixel. Nevertheless, several vegetation indices derived from ABI surface reflectances serve as reliable proxies for GPP. Across all sites, ecosystem types, and times of day, the r^2^ values between GPP and NDVI, NIRv, and NIRvP derived from ABI are respectively 0.337, 0.355, and 0.420. To compare half-hourly ABI and tower data to once-daily MODIS observations, we computed “midday means” by averaging observations over a 2.5 hour midday window centered at the time of that day’s solar zenith. The midday NIRv indices derived from ABI and a MODIS eight-day surface reflectance product have a strong positive correlation with r^2^ = 0.705. When using the midday ABI indices to predict midday GPP, the r^2^ values are 0.405, 0.426, 0.507 for NDVI, NIRv, and NIRvP. However, MODIS NIRv is a slightly stronger predictor of midday GPP with r^2^ = 0.586, likely because its spatial resolution is four-times higher than ABI^64^ at nadir and because the reflective channels used have slightly different bandwidths.

To further validate our results, we evaluated on a site-by-site basis key variables related to carbon cycling that vary by ecosystem type. We first describe patterns that emerge when investigating data from pixels that include all 314 tower sites, then describe time series from six different ecosystems that reflect a range of the different conditions encountered in the dataset. The ecosystems are a dry forest (BR-CST) and a wetland (PE-QFR) in the South American Tropics, and a corn cropland (US-Br1), grazed grassland (US-CGG), deciduous broadleaf forest (US-Cwt) and needleleaf evergreen forest (US-Ho1) in the United States (see *Site descriptions for six sample AmeriFlux sites* in the Supplementary Information^65–72^).

**Fig. 8:**
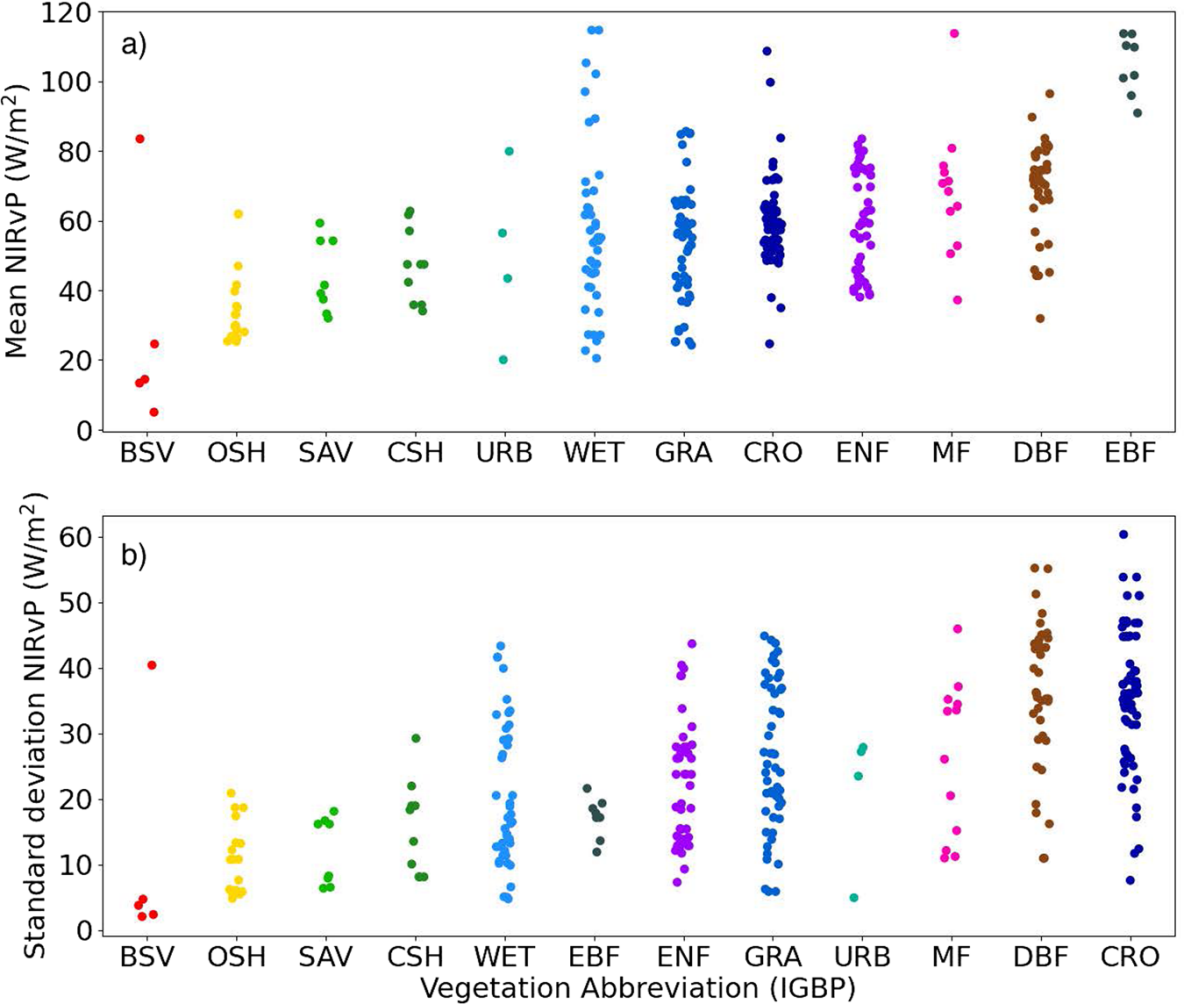
Strip plots showing the mean (top) and standard deviation (bottom) of the average midday NIRvP across each month of the year. Midday NIRvP, the near infrared reflectance of vegetation multiplied by photosynthetically active radiation (units W m-2), is the median value between 10:00 and 14:00 local time. Sites are sorted by the International Geosphere Biosphere Programme (IGBP) vegetation type^74^ at the location of the eddy covariance towers.

Figure 8 illustrates the variability in midday NIRvP across ecosystem types. NIRvP generally increases in the morning and decreases in the afternoon due to the DSR (Fig. 5, 6), causing the diurnal peak to occur around midday. We generated daily NIRvP values by calculating the median from all the observations within the midday window (between 10:00 and 14:00 local standard time) at every site. Then, at each site, we calculated monthly means by averaging all the daily NIRvP values over the month, creating summarized chronologies of NIRvP behavior throughout the year (Fig. 8a). Deciduous and especially evergreen broadleaf forests have the highest average NIRvP year-round, and barren ecosystems and open shrublands have the lowest. Wetland ecosystems have the highest between-site variability in NIRvP. The NIRvP standard deviation figure (plotting standard deviations of the monthly midday medians) highlights which land cover types vary most strongly across the data record (Fig. 8b). The monthly standard deviation in NIRvP tends to be relatively low when its mean is also low. On the other end, croplands and deciduous broadleaf sites have high standard deviation in NIRvP, as expected due to the highly seasonal nature of these vegetation types. More insight into the temporal dynamics of different ecosystem types can be gained by a close examination of representative examples.

**Fig. 9:**
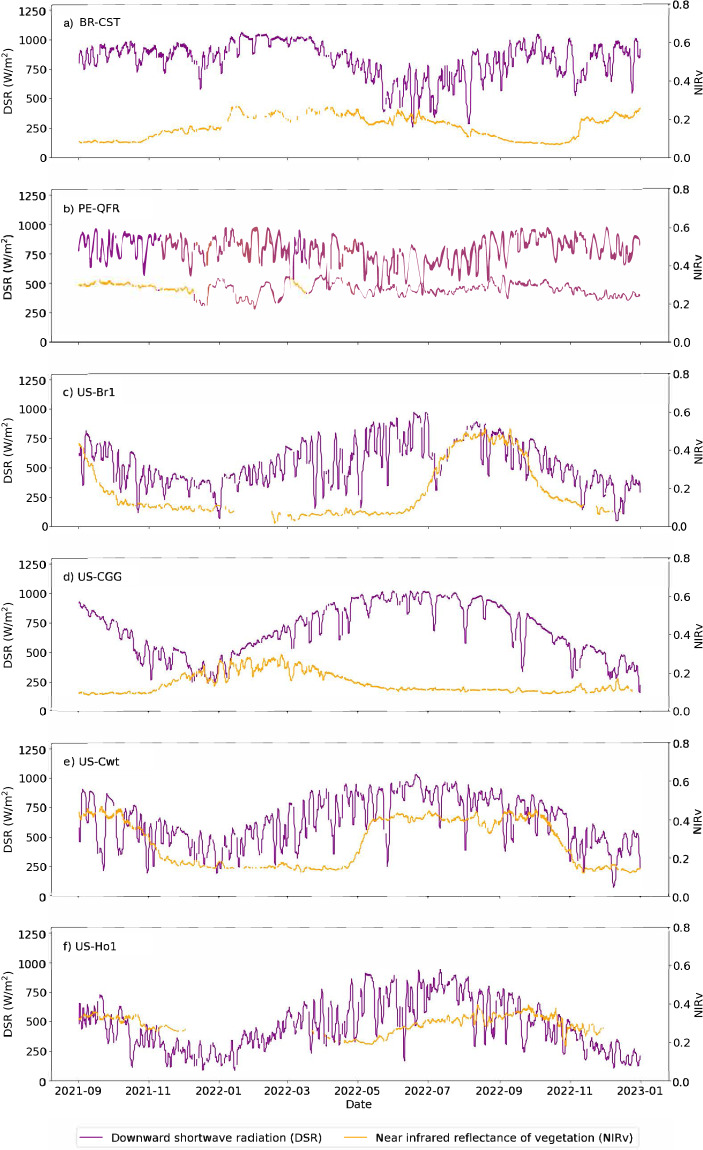
Fourteen-day moving averages of the downward shortwave radiation (DSR) and the near infrared reflectance of vegetation (NIRv) for the six representative AmeriFlux eddy covariance tower sites described in the text for the late August 2021 - December 2022 period for which the ABI surface reflectance product was available. DSR values were removed if their corresponding DQFs were equal to 1, signaling an invalid or degraded observation. NIRv observations were removed where there was snow cover, as seen during the winter periods of site US-Ho1 (**f**). Pixels with snow cover were masked using a DQF from the Aerosol Detection product.

The time series of DSR and NIRv over the sixteen-month period for which GOES-R surface reflectance observations were available elucidate site-specific trends in climate and phenology (Fig. 9). Fourteen-day moving averages help encapsulate the signal while demonstrating the intra-daily variability. Equatorial sites (Fig. 9a,b) receive more consistent solar radiation year-round, but cloud cover is more likely to interfere during the wet season (May through August) at BR-CST. The wetland PE-QFR is moderated by the water-saturated substrate, causing steadier NIRv – and therefore likely vegetation productivity – than at BR-CST, a tropical dry forest. The other four Northern Hemisphere sites (Fig. 9c–f) receive higher solar insolation in the summer, but phenological trends are land cover dependent. The evergreen forest (US-Ho1) has higher NIRv on average than the deciduous forest (US-Cwt) which surges in productivity during spring leaf-up and declines during fall senescence; the missing data in the winter at US-Ho1 is due to snow cover. Compared to these natural forests, the corn crop at US-Br1 has a more dramatic and condensed growing season. In the Mediterranean climate of the US-CCG site, the grass NIRv reaches its highest values in the cool winter season.

**Fig. 10:**
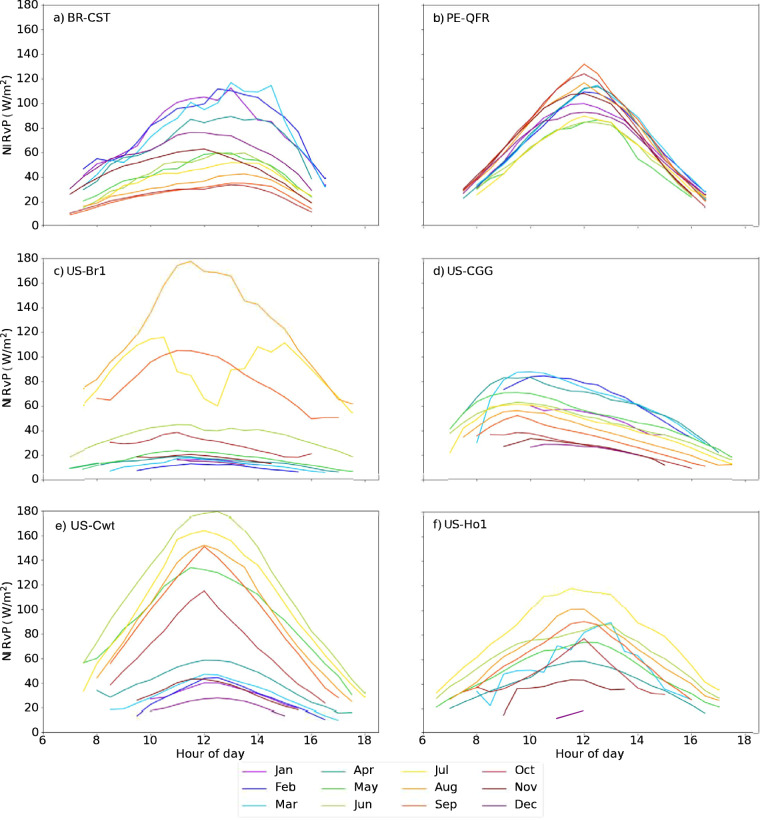
The diurnal fluctuations of GOES-R NIRvP by month for the six representative AmeriFlux eddy covariance sites studied here. Before averaging the midday peaks in NIRv, surface reflectance pixels covered by snow and invalid DSR pixels were removed according to the Aerosol Detection and DSR DQFs. The BRF product only generates reflectance values when the solar zenith angle (SZA) is less than or equal to 67 degrees, which eliminates entire months of data at high latitudes like at US-Ho1 (**f**).

While Fig. 9 points to yearly variability in DSR and NIRv, Fig. 10 depicts how NIRvP – the product of DSR and NIRv with the former adjusted to approximate PAR – fluctuates on average over the course of a single day. Hourly mean NIRvP values are further broken down by month, representing how landscapes exhibit unique diurnal patterns at different times of year. The general form of this pattern is a midday peak in NIRvP due to DSR reaching its peak at the daily solar zenith. July at the US-Br1 cropland (Fig. 10c) is a notable exception, likely caused by midday clouds. At the wetland PE-QFR (Fig. 10b), the diurnal NIRvP pattern stays consistent between months while the deciduous forests and agricultural field show striking differences between seasons. At a couple of sites, the maximum NIRvP does not coincide with solar noon, creating asymmetrical curves. The BR-CST peak shifts towards the afternoon while US-CGG shifts towards the morning (Fig. 10a,d).

**Fig. 11:**
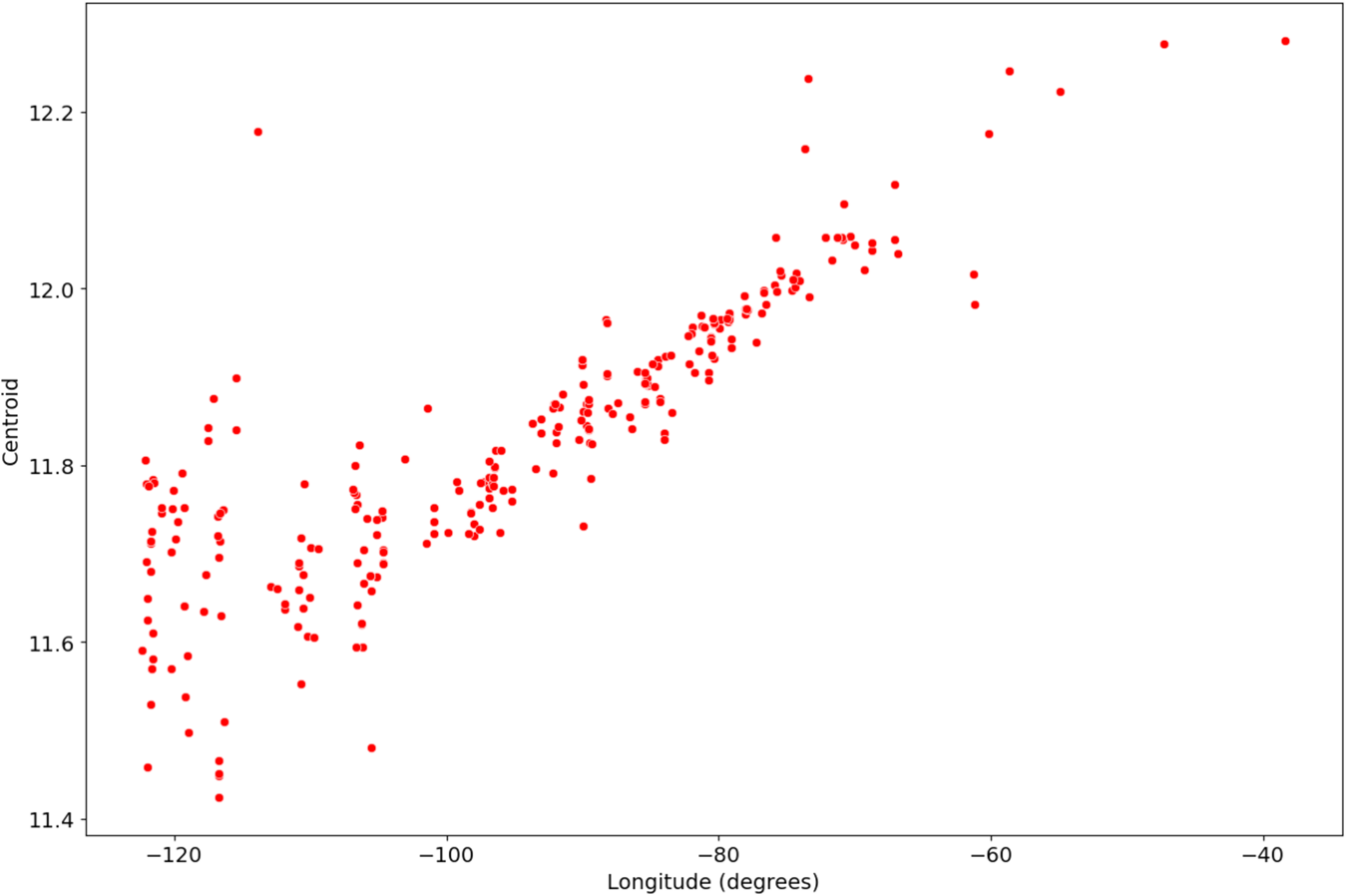
The diurnal centroid of NIRvP from GOES-16 for all 314 AmeriFlux eddy covariance tower locations.

This diurnal asymmetry is driven in part by NIRv, which can be attributed to the site-specific viewing geometry – the site longitude (related to the satellite’s VZA) is tightly correlated to diurnal asymmetry (Fig. 11). In other words, the further a site is from the GOES-16 sub-satellite point (75.2 degrees East), the more the reflectance peak strays from solar noon. Other factors contributing to the asymmetry, such as biological properties of the ecosystem, are site-dependent and require further study. To measure asymmetry, we calculated the diurnal centroid at each site by taking the mean diurnal time weighted by the half-hourly mean NIRvP by month (Eq. 8) (ref. ^73^). Hence, any deviation from 12 (local noon) represents a shift towards the morning or afternoon. Since the vast majority of our sites are West of GOES-16, it is logical that most sites would have a diurnal centroid under 12 corresponding with a morning shift in peak NIRvP.

8 $$centroid=\frac{{\sum }_{t=0}^{24}NIRv{P}_{t}\times t}{{\sum }_{t=0}^{24}NIRv{P}_{t}}$$

## Usage Notes

Knowing the value of the environmental variable is only useful if the pixel’s data is trustworthy and credible. Provided alongside each included ABI product, the corresponding “data quality flag” (DQF) offers a reliability label for every pixel. From DQFs we can learn whether the product algorithm ran successfully, whether the input data was high quality, and the confidence level of the output environmental metric. Furthermore, DQFs explain the conditions under which the pixel was acquired, like the solar and viewing geometry, and additional information about the target like the land cover type or cloud cover. These DQFs can be applied to time series at the users’ discretion.

There are two types of DQFs in this dataset: simple categorization and bit masks. For the former, the value of the DQF falls into just one category, such as ‘high quality’ or ‘low quality.” The CMI, Clear Sky Mask, AOD, and DSR products are tagged with simple categorical DQFs (Table S3). The other four products – BRF, LSA, Aerosol Detection, and LST – use bit masks, which offer more nuance and rationale behind the DQF designation (Table S4). Eight-bit masks provide eight binary pieces of information about the data quality (each bit is equal to 0 or 1). In this way the DQF can offer important context, for example, if the pixel has high quality input data, but the surface is obstructed from view by an opaque cloud. An individual bit equal to 0 rather than 1 typically means that the data is high quality, or the retrieval was successful. Before using any environmental variable time series, we recommend filtering out poor quality and irrelevant observations using the DQFs. We will describe one product, BRF, to illustrate how to take advantage of the rich informational content contained in the DQFs.

Each ABI product has a unique DQF convention; it is advised to familiarize oneself with all the unique DQF values and meanings associated with each product. Table 3 shows the eight possible DQF bit masks for the BRF product. When using bit masks, we recommend first checking which DQF values are present in the dataset, then using our DQF reference tables to interpret their significance (see Tables S3, S4 for the other ABI products). For the BRF product, the BRF_DQF flags are one of four unique values: 8, 16, 24 or 26. By converting these integers to binary, we learn which bits are set to 1 and which are set to 0 (Table 4). In this case, we see that the only bits set to 1 in any of these binary values are bits 1, 3 and 4 (respectively 1, 3 or 4 places from the leftmost digit). We will use Table 3 to explain what these bit masks mean. The solar zenith angle (SZA) varies throughout the day and the algorithm only runs during daylight when SZA < 67 degrees (bit mask 1). When SZA ≥ 67, it is considered nighttime because the algorithm considers the sunlight levels to be too low. The retrieval path flag (bits 3 and 4) declares which method was used to calculate the surface reflectance value, algorithm R2 (when clear-sky) or R3 (when cloudy), as previously described in the *Methods* section. To put all the pieces together, if your application is only concerned with clear-sky observations during the day, the BRF data should only be used when the BRF DQF pixel values are equal to 8.

**Table 3:** The eight bits and their flag name and meanings for the Bidirectional Reflectance Factor (BRF) data quality flag (DQF) variable.

Bit	Flag Name	Flag Value
0	Land mask	0: Land, 1: Water
1	SZA	0: SZA < 67, 1: SZA 67
2	VZA	0: VZA < 70, 1: VZA 70
3	Retrieval path	00: R1, 01: R2
4		10: R3 11: At least one band has no retrieval
5	Cloud information	00: Absolutely clear, 01: Probably clear
6		10: Probably cloudy, 11: Absolutely cloudy
7	Empty

**Table 4:** The interpretation of the four bit masks actually present in the BRF DQF data (8, 16, 24, 26), ascertained by sectioning the bit mask into the binary values of individual bits, and using Table 3 to determine the meaning of each flag value.

Bit mask	8-bit binary	Bit 1	Bits 3 and 4	Flag values	Interpretation
8	00001000	0	01	R2 retrieval path, SZA < 67	Clear-sky retrieval, daytime
16	00010000	0	10	R3 retrieval path, SZA < 67	Cloudy retrieval, daytime
24	00011000	0	11	No retrieval, SZA < 67	No retrieval, daytime
26	00011010	1	11	No retrieval, SZA > 67	No retrieval, nighttime

With this understanding of how the bit masks work, we will discuss the implications of the BRF quality flags (Table 3) to exemplify the limitations of ABI products. Data availability is limited spatially and temporally because the BRF algorithm is dependent on viewing geometry and land cover. The BRF algorithm will not run over water because its reflective character is different from land (bit mask 0). The present dataset does not include any sites that have water rather than land cover. There are no valid measurements when either the sun or satellite stray significantly from the zenith, the highest point in the sky relative to the surface target. The VZA of a geostationary satellite to a target on the surface does not change, hence the geographical range where data gets processed is always limited to VZA < 70 degrees (bit mask 2). For example, the GOES-16 full disk BRF product is valid across most of the continental United States, but excludes the northwestern US, Alaska, and central-northwestern Canada (Fig. 3). The SZA, however, changes constantly, which makes bit mask 1 important for quality control and affects the amount of data availability by site. In the Northern Hemisphere winter when the sun is low in the sky and daylight is short-lived, BRF data are limited to a few mid-day measurements (e.g. Figure 10c–e), or to none at all at high latitudes. Inversely, long summer days at high latitudes have more BRF measurements than lower latitudes due to the advantageous sun angles. Near the equator, the number of BRF measurements per day is much less variable (see e.g. Figure 10a,b).

**Fig. 12:**
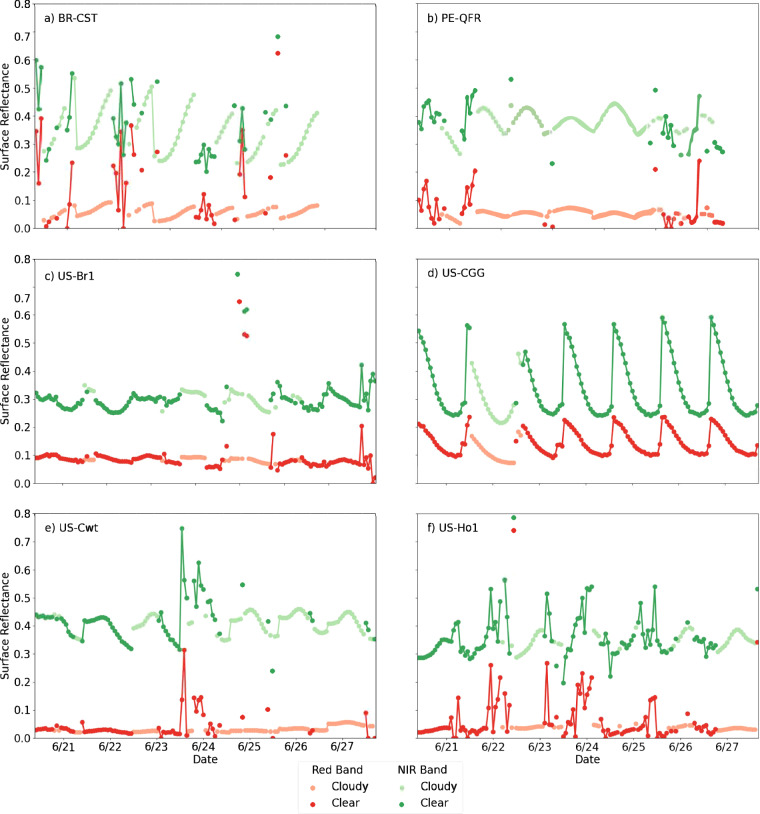
Surface reflectances in the red (ABI band 2) and near infrared (ABI band 3) for the six example eddy covariance sites described in Technical Validation during a one-week period in June. The hue of observations indicates whether the clear-sky or cloudy algorithm (R2, dark hue or R3, light hue) was implemented.

Bit masks 3 and 4 contain information about the algorithm retrieval path, which may have a significant effect on the data content. The user will decide whether their final application requires filtering BRF data on this criterion. Retrieval paths R2 and R3 derive the surface reflectances in different ways, using direct observations when the sky is clear (R2) and modeling from prior observations when it is cloudy (R3). BRF reflectances, sorted by retrieval path, are plotted in Fig. 12 at six sites over one week in June. Figure 12 makes apparent the variability of clear-sky observations – the signal can be extremely noisy or very smooth depending on the site and date. Compare, for example, the smooth diurnal curves at US-CGG (California) to the jagged spikes at US-Ho1 (Maine). We theorize that noisy clear-sky time series (R2) are the result of thin undetected clouds and aerosols obscuring the surface reflectance signal and sub-pixel variability. On the other hand, the modeled surface-reflectance measurements under cloudy-sky conditions (R3) are consistently smooth. These estimates, however, have more uncertainty especially following long stretches without clear-sky observations because they rely on prior clear-sky observations to extrapolate the reflectance under the clouds. Bit masks 5 and 6 would describe the likelihood of cloudiness for each pixel, however, these bits are not currently being used in the processing stream, nor is the “empty” bit mask 7. Empty or unused bit masks are always set to 0.

In summary, GOES-R data can provide rich time series that helps quantify the variability in key land surface attributes that are related to carbon cycling, but the native data format and challenges with terrain-correction have limited its ability to link with surface-atmosphere fluxes like the eddy covariance time series organized by AmeriFlux, NEON Inc. and others that are used by the carbon cycle community. By providing GOES-R observations for 314 eddy covariance sites we hope to provide a way forward to better link hypertemporal and sub-daily satellite and eddy covariance observations.

### Supplementary information

Supplementary Information

Supplementary table S2

## Data Availability

Code is available on Google Colab at: https://colab.research.google.com/drive/1lgyPhYVXr4MffWnN7m-5Bo3Lt5f4f49D?usp=sharing.
