# Supplementary material for: GOES-R land surface products at Western Hemisphere eddy covariance tower locations
Source: Sci Data. 2024 Mar 7;11:277. doi: 10.1038/s41597-024-03071-z (PMC10920807; doi:10.1038/s41597-024-03071-z)
Supplement: Supplementary file 1 — Supplementary Information [file 41597_2024_3071_MOESM1_ESM.docx]

### Supplementary Information

*Converting between projections*

The geodetic latitudes and longitudes of AmeriFlux towers are converted to ABI scan angles (with a parallax adjustment applied) to geolocate the towers on the ABI Fixed Grid (Equations S1 - S7). Other earth science applications will often do the inverse operations to convert the satellite's scan angles to geodetic latitudes and longitudes (Equations S8 - S15). The L2 DSR product is not projected on the ABI fixed grid, but instead uses the Global Latitude and Longitude, also known as the equirectangular projection. However, the eddy covariance site geodetic coordinates still must be adjusted to account for the parallax effect. Therefore, the ABI scan angles are computed from the site latitude, longitude and elevation, then converted back to geodetic latitude and longitude, using both sets of equations below (Equations S1 - S15).

All of the equations below are described in greater detail in the GOES-R PUG Volume 5, Section 4.2.8 “Navigation of Image Data,” pages 21 - 26. Figure 4.2.8 illustrates the GRS 80 ellipsoid Earth model and the relationship between the two coordinate frames/projections. In the two sets of equations below, some of the same physical parameters are defined differently depending on which values are unknown (unknowns are either latitude/longitude or ABI scan angles). Please refer to Figure 4.2.8 in the GOES-R PUG Volume 5 for the entire visualization.

*Geodetic latitude and longitude to ABI scan angles*^38^

The N/S elevation angle (*y*) and E/W scanning angle (*x*) are computed using

$y = arctan\left( \frac{s_{z}}{s_{x}} \right)$ (S1)

$x = arcsin\left( \frac{-s_{y}}{\sqrt{s_{x}^{2}+s_{y}^{2}+s_{z}^{2}}} \right)$ (S2)

Where

$s_{x}=H-r_{C}cos\left( \phi_{C} \right)cos\left( \lambda-\lambda_{0} \right)$ (S3)

$s_{y}=-r_{C}cos\left( \phi_{C} \right)sin\left( \lambda-\lambda_{0} \right)$ (S4)

$s_{z}=r_{C}sin\left( \phi_{C} \right)$ (S5)

H is the height of the satellite from the center of the Earth (4,2164,160 m), $\lambda_{0}$ is the longitude of the projection origin (−1.309 radians), $\phi$ is the GRS80 geodetic latitude in radians, $\lambda$ is the geodetic longitude in radians, the geocentric latitude is:

$\phi_{C}=arctan\left( \frac{r_{pol}^{2}}{r_{eq}^{2}}tan\left( \phi\right) \right)$ (S6)

And the geocentric distance to the point on the ellipsoid is
 $r_{C} = \frac{r_{pol}}{\sqrt{1-e^{2}{cos}^{2}\left( \phi_{C} \right)}}$ (S7)

*ABI scan angles to geodetic latitude and longitude*[*^38^*](https://www.zotero.org/google-docs/?rMhpxJ)

$\phi= arctan(\frac{{r_{eq}^{2}s}_{z}}{r_{pol}^{2}\sqrt{{(H - s_{x})}^{2}+s_{y}^{2}}})$ (S8)

$\lambda= \lambda_{0} - arctan (\frac{s_{y}}{H-s_{x}})$ (S9)

Where

$r_{s}= \frac{-b-\sqrt{b^{2}-4ac}}{2a}$ (S10)

In which

$a = {sin}^{2}(x) + cos^{2}(x) (cos^{2}\left( y \right)+\frac{r_{eq}^{2}}{r_{pol}^{2}} sin^{2}\left( y \right))$ (S11)

$b = -2Hcos(x)cos(y)$ (S12)

$c = H^{2}-r_{eq}^{2}$ (S13)

$s_{x}=r_{s}cos(x)cos(y)$ (S14)

$s_{y}= -r_{s}sin(x)$ (S15)

Table S1. Band characteristics– central wavelength and spatial resolution at nadir– for the 16 ABI channels.

| **ABI Band** | Wavelength  (μm) | Resolution (km) | **ABI Band** | Wavelength  (μm) | Resolution (km) |
| --- | --- | --- | --- | --- | --- |
| **1** | 0.47 | 1 | **9** | 6.9 | 2 |
| **2** | 0.64 | 0.5 | **10** | 7.3 | 2 |
| **3** | 0.86 | 1 | **11** | 8.4 | 2 |
| **4** | 1.37 | 2 | **12** | 9.6 | 2 |
| **5** | 1.6 | 1 | **13** | 10.3 | 2 |
| **6** | 2.2 | 2 | **14** | 11.2 | 2 |
| **7** | 3.9 | 2 | **15** | 12.3 | 2 |
| **8** | 6.2 | 2 | **16** | 13.3 | 2 |

*EDI Data Package Summary*

The Environmental Data Initiative (EDI) is an online data repository with resources to help archive and publish high-quality environmental and ecological data and metadata in a manner that ensures the data are well curated and accessible for discovery and re-use.

Each data package includes a summary describing basic publication information such as authors, citations, package identifiers, intellectual rights, and an overall abstract, as well as a full metadata and quality report with file sizes for each dataset, and the total number of views and downloads by other people. The data package also includes the option to download or explore the datasets, or to export the metadata into different coding language formats including: Matlab, Python, R, SAS, SPSS, and tidyr.

The detailed metadata includes further information such as data package usage rights, keywords, temporal and geographic coverage, collection methods and protocols, data provenance which notes where the processed data is derived from, people and organizations involved, and maintenance. Methods and protocols used in the collection of this data package are described in detail in the methods section of this manuscript. The basic metadata for each data table includes information such as: column names and definitions, number of rows and columns, storage type, measurement and unit type, missing value codes, an accuracy assessment, and the overall spatial and temporal coverage.

Given the large size and number of files, our full dataset is provided in a .zip format, therefore the metadata for the 20-22_GOES-R_AmeriFlux_datasets is described in the data tables “example_file” and the AmeriFlux site information for all 314 sites is detailed in “GOES-R_AmeriFlux_site_info” (Table S2), available at:

<https://doi.org/10.6073/pasta/c3bb20a62edbf8548cbb30e79a689a5b>^62^.

Table S2: *GOES-R_AmeriFlux_site_info.csv* (0.031 MB)

The site identifier, tower locations including parallax correction, and climate and ecosystem type of the 314 AmeriFlux eddy covariance sites are cataloged here. Upon re-collection, 318 sites were available in the growing AmeriFlux database and are made available for completeness. Each active AmeriFlux tower site is reported with its respective site information in the following 12 fields:

Site ID, local time zone, view zenith angle (VZA) and view azimuth angle (VAA) to GOES-16, parallax displacement, elevation, ABI pixel area, latitude and longitude, terrain-corrected latitude and longitude, Koeppen climate classification, and IGBP land cover classification.

The following zip files contain unique files for each AmeriFlux tower site location, divided alphabetically with NEON and international sites separated out.

*20-22_GOES-R_AmeriFlux_datasets_international.zip* (352 MB)

*20-22_GOES-R_AmeriFlux_datasets_NEON.zip* (284 MB)

*20-22_GOES-R_AmeriFlux_datasets_A-G.zip* (518 MB)

*20-22_GOES-R_AmeriFlux_datasets_H-O.zip* (530 MB)

*20-22_GOES-R_AmeriFlux_datasets_P-W.zip* (629 MB)

Each file includes an observation at every timestamp over the 63 fields defined in Table 1, Table S3, Table S4 and in the text, as summarized below.

*Viewing geometry and time*:  UTC timestamp, local timestamp, day of year, hour of day, solar zenith angle (SZA), solar azimuth angle (SAA), and solar position (SZA + SAA).

*GOES-R Series ABI product variables and quality flags (DQFs)*: Cloud and moisture imagery (CMI) bands 1-16, Bidirectional Reflectance Factor (BRF) for bands 1-3, 5, and 6, Land Surface Albedo (LSA), Clear Sky Mask (ACM), Aerosol Optical Depth (AOD, Aerosol Detection masks for aerosols, smoke, and dust, Land Surface Temperature (LST), and Downward Shortwave Radiation (DSR).

*Derived products*:  Normalized difference vegetation index (NDVI), Near infrared reflectance of vegetation (NIRv), Photosynthetically active radiation (PAR), and NIRv multiplied by PAR (NIRvP).

The temporal and geographic coverage of datasets are as follows. The time period covers 2020-01-01 to 2022-12-31, with the exception of the BRF and LSA products which begin their availability on 2021-08-21.

There are 318 eddy covariance tower locations from the AmeriFlux and NEON tower networks spread throughout North and South America, contained within the GOES-16 full disk coverage (Figure 1).

West: (0°, -156.30°) East: (0°, 6.30°) North: (81.33°, 0°) South: (-81.33°, 0°)

Altitude extent: -53.0 meters to 3513.0 meters

*Site descriptions for six sample AmeriFlux sites*

*BR-CST*

Caatinga Serra Talhada (BR-CST) is a tropical dry forest on the far eastern side of Brazil^67^. The vegetation is deciduous needleleaf forest, but open enough to allow for cattle grazing in the wet season. The semi-arid Steppe climate delivers cold winter temperatures. No logging has taken place here for at least 50 years.

*PE-QFR*

Quistococha Forest Reserve (PE-QFR) is a tropical peatland palm swamp in northeastern Peru^68^. The site is just outside the city of Iquitos in a natural protected forest reserve, and also nearby the Amazon River. The predominant vegetation is *Mauritia flexuosa*, a wetland palm. The tropical climate here is defined by a long wet season and short dry season from June to August^69^.

*US-Br1*

Brooks Field Site 10-Ames is a cropland that rotates between corn and soy, depending on the year^70^. The site is in Ames, Iowa, just north of Des Moines in the heart of the Upper Midwest Corn Belt. The humid continental climate is characterized by very cold winters, hot summers, and year-round precipitation. Two additional eddy covariance towers are located at different fields on the same farm named US-Br2 and US-Br-3.

*US-CGG*

The Concord Grazed Grassland (US-CGG) rangeland is tucked into the suburbs of Concord, California, within the East Bay Area. While the property is part of California State University’s East Bay Concord Campus, the grassland is managed by a local rancher who grazes around 60 cattle in the cool season from December to April. During the cool season, temperatures remain mild and annual grasses dominate due to the Mediterranean climate.

*US-Cwt*

Coweeta (US-Cwt) is a southern Appalachian site at an elevation of 690 meters in western North Carolina near the border with Georgia, near the USFS Coweeta Hydrologic Laboratory^71,72^. This temperate secondary forest is primarily deciduous broadleaf, and the Warm Summer Continental climate exhibits significant rainfall year-round. The forest was logged until the 1930s.

*US-Ho1*

Howland Forest is an evergreen needleleaf forest in central Maine and the US-Ho1 eddy covariance tower has one of the longest flux records dating back to 1996 (ref. 73). The Howland Research Forest was founded by a partnership between the University of Maine and the International Paper Company. Stands are multi-aged due to its commercial history of logging select species. The forest is dominated by spruce, hemlock and fir and lies at the transition between northeastern deciduous forest and boreal evergreen forest^74^. Two other towers, US-Ho2 and US-Ho3 are also located at Howland Forest.

Table S3. Categorical data quality flags (DQFs).

| **Flag Value** | **Flag Meaning** |
| --- | --- |
| **Cloud and Moisture Imagery (CMI)** | |
| **0** | Good quality pixel |
| **1** | Conditionally usable pixel |
| **2** | Out of range pixel |
| **3** | No value pixel |
| **4** | Focal plane temperature threshold exceeded |
| **Clear Sky Mask** | |
| **0** | Good quality pixel |
| **1** | Invalid due to improper geolocation or algorithm failure |
| **2** | Degraded due to VZA threshold exceeded |
| **3** | Invalid due to bad or missing data for 11.9m band |
| **4** | Degraded due to bad data from 3.89 m band |
| **5** | Degraded due to failed tests on 0.64m band |
| **6** | Degraded due to other bad bands |
| **Downward Shortwave Radiation (DSR)** | |
| **0** | Good quality pixel |
| **1** | Degraded quality or invalid |
| **Aerosol Optical Depth (AOD)** | |
| **0** | High quality retrieval |
| **1** | Medium quality retrieval |
| **2** | Low quality retrieval |
| **3** | No retrieval |
| **Land Surface Temperature (LST) on August 20th, 2021 and after** | |
| **0** | High quality retrieval |
| **1** | Medium quality retrieval |
| **2** | Low quality retrieval |
| **3** | No retrieval |

Table S4. Bit mask data quality flags (DQFs).

| **Bit** | **Flag Name** | **Flag Value** |
| --- | --- | --- |
| **Bidirectional Reflectance Factor (BRF)** | | |
| 0 | Land mask | 0: Land,                                1: Water |
| 1 | SZA | 0: SZA < 67,                        1: SZA 67 |
| 2 | VZA | 0: VZA < 70,                       1: VZA 70 |
| 3 | Retrieval path | 00: R1,                               01: R2 |
| 4 |  | 10: R3                                11: At least one band has no retrieval |
| 5 | Cloud information | 00: Absolutely clear,         01: Probably clear |
| 6 |  | 10: Probably cloudy,         11: Absolutely cloudy |
| 7 | Empty |  |
| **Land Surface Albedo (LSA)** | | |
| 0 | Land mask | 0: Land,                              1: Water |
| 1 | SZA | 0: SZA < 67,                      1: SZA 67 |
| 2 | LZA | 0: SZA < 70,                      1: SZA 70 |
| 3 | Retrieval path | 00: Routine algorithm,     01: Back-up algorithm |
| 4 |  | 10: Graceful degradation, 11: No retrieval |
| 5 | AOD quality | 00: High quality,               01: Medium quality |
| 6 |  | 10: Low quality,                11: No retrieval |
| 7 | Empty |  |
| **Aerosol Detection** | | |
| 0 | Smoke detection retrieval | 0: Good retrieval,              1: Invalid due to snow, ice, clouds or degraded data |
| 1 | Dust detection retrieval | 0: Good retrieval,              1: Invalid due to snow, ice, clouds or degraded data |
| 2 | Smoke detection confidence | 00: Low confidence,        01: Medium confidence |
| 3 |  | 11: High confidence |
| 4 | Dust detection confidence | 00: Low confidence,        01: Medium confidence |
| 5 |  | 11: High confidence |
| 6 | Sun glint | 0: Out of sun glint,            1: Within sun glint |
| 7 | SZA and VZA | 0: Within valid range,       1: Outside of valid SZA and VZA range |
| **Land Surface Temperature (LST) before August 20th, 2021** | | |
| 0 | Empty |  |
| 1 | Availability | 0: Valid input data,           1: Invalid due to bad or missing input data |
| 2 | Cloud index | 0: Valid clear conditions,  1: Invalid due to cloudy conditions |
| 3 | VZA | 0: Valid VZA,                   1: Degraded due to VZA threshold exceeded |
| 4 | Surface type | 0: Land or inland water,   1: Invalid to to water surface type |
| 5 | LST quality | 0: Valid LST,                    1: Invalid due to LST out of range (not in 213-330K) |
| 6 - 7 | Empty |  |
